# Development of novel artificial intelligence systems to predict facial morphology after orthognathic surgery and orthodontic treatment in Japanese patients

**DOI:** 10.1038/s41598-021-95002-w

**Published:** 2021-08-04

**Authors:** Chihiro Tanikawa, Takashi Yamashiro

**Affiliations:** 1 Department of Orthodontics and Dentofacial Orthopedics, Graduate School of Dentistry, Osaka University, Suita, Osaka Japan; 2 Center for Advanced Medical Engineering and Informatics, Osaka University, Suita, Osaka Japan; 3 Institute for Datability Science, Osaka University, Suita, Osaka Japan

**Keywords:** Computer science, Orthodontics, Craniofacial orthodontics, Fixed appliances, Occlusion, Diagnosis

## Abstract

From a socio-psychological standpoint, improving the morphology of the facial soft-tissues is regarded as an important therapeutic goal in modern orthodontic treatment. Currently, many of the algorithms used in commercially available software programs that are said to provide the function of performing profile prediction are based on the false assumption that the amount of movement of hard-tissue and soft-tissue has a proportional relationship. The specification of the proportionality constant value depends on the operator, and there is little evidence to support the validity of the prediction result. Thus, the present study attempted to develop artificial intelligence (AI) systems that predict the three-dimensional (3-D) facial morphology after orthognathic surgery and orthodontic treatment based on the results of previous treatment. This was a retrospective study in a secondary adult care setting. A total of 137 patients who underwent orthognathic surgery (n = 72) and orthodontic treatment with four premolar extraction (n = 65) were enrolled. Lateral cephalograms and 3-D facial images were obtained before and after treatment. We have developed two AI systems to predict facial morphology after orthognathic surgery (System S) and orthodontic treatment (System E) using landmark-based geometric morphometric methods together with deep learning methods; where cephalometric changes during treatment and the coordinate values of the faces before treatment were employed as predictive variables. Eleven-fold cross-validation showed that the average system errors were 0.94 mm and 0.69 mm for systems S and E, respectively. The total success rates, when success was defined by a system error of < 1 mm, were 54% and 98% for systems S and E, respectively. The total success rates when success was defined by a system error of < 2 mm were both 100%. AI systems to predict facial morphology after treatment were therefore confirmed to be clinically acceptable.

## Introduction

The face plays an important role as a means of nonverbal communication in the transmission of emotions and thoughts in social life. Further, the degree of recognition of an individual's face by others has a significant socio-psychological impact on the acceptance of the individual in their community. If there is dysmorphology of the maxillofacial region, such as cleft palate, or morphological distortion of the face due to jaw deformity, it may cause serious socio-psychological maladaptation. From this socio-psychological standpoint, improving the morphology of facial soft-tissues is regarded as an important therapeutic goal in modern orthodontic treatment^1^. When planning treatment for patients with malocclusion, the prediction of profile and/or facial changes that will occur after treatment is of the utmost importance when judging whether a treatment method is appropriate (i.e., tooth extraction or non-extraction, treatment with or without orthognathic surgery).

Thus far, many studies have tried to predict facial profiles after orthodontic treatment and have examined the facial changes according to orthodontic treatment (i.e., four premolar extraction^2–11^, non-extraction treatment^7,8,10–12^, orthognathic surgery^13–15^, or the use of a functional appliance^16–18^). As for the profile changes that occur following four-premolar extraction, most studies have evaluated the relationship between incisor movement and soft-tissue profiles on cephalograms^2–6^. Previous studies examining factors associated with two-dimensional (2-D) profile changes after orthodontic treatment have revealed a proportional relationship between the amount of movement of hard and soft-tissues. However, the value of the proportionality constant in the lip region has been shown to vary greatly depending on the case^19–21^. Currently, many of the algorithms used in commercially available software programs that are said to provide the function of performing 2-D profile prediction are based on the false assumption that the amount of movement of hard-tissue and soft-tissue has a proportional relationship. The specification of the proportionality constant value depends on the operator, and there is little evidence regarding the validity of the prediction result. Furthermore, regarding the pattern matching technology used, the specific algorithm has not been clarified, and it is unclear whether a valid prediction has been made^22^. According to studies^22–26^ that compared results predicted with such software programs to actual treatment results, it is reported that it is still difficult to predict the facial morphology before and after orthodontic treatment.

Recently, three-dimensional (3-D) stereophotogrammetry of faces has emerged in dentistry and orthopedics as a method of recording 3-D facial topography^27–31^. Using this technique, 3-D images are acquired by combining photographs captured from various angles with synchronous digital cameras. The values recorded by 3-D systems are reported to be accurate and reliable for clinical use^27,28^. Since 3-D data include a greater amount of information than two-dimensional data, it was assumed that 3-D images are useful for creating a system that predicts the post-treatment facial shape. Although commercial 3-D simulation software programs exist (e.g., Dolphin system), they are only for prediction after orthognathic surgery and internal calculations have not been clarified; thus it is unclear whether their calculations are based on evidence. Studies that measured the ability to accurately predict 3-D soft-tissue changes after Le Fort I osteotomy and/or sagittal split osteotomy using the Dolphin 3-D software program^32,33^ showed limited accuracy, especially in the upper lip and base of the nose. There is a great need for patients to be able to check the post-treatment face in 3-D before treatment; thus, a 3-D facial simulation system that can predict 3-D faces after orthognathic surgery or orthodontic treatment with four premolar extraction would be a clinically important tool.

On the other hand, with recent computation advances, two important methods that are related to 3-D prediction have become available in machine learning, that is, landmark-based geometric morphometric methods (GMMs) and deep learning. GMMs that have revealed some statistical variation in the shape and size of target objects (phenotypic variation) have recently emerged. In developmental biology, GMMs use homologous landmarks, which can be defined as precise locations on biological specimens that hold some functional, structural, developmental, or evolutionary significance, and which are directly comparable between specimens^34^. A key concept of a GMM is based on the fact that the morphology can be systematically mapped, often within a “morphospace”, with the use of these landmarks. Morphospaces are maps that show how shapes are defined by quantitative traits. GMMs rely on the superimposition of landmark coordinate data to place individuals in a common morphospace. As deep learning should use the same number of inputs, we hypothesized that this GMM would be useful, together with deep learning methods, for developing artificial intelligence (AI) systems that predict the 3-D facial topography after orthognathic surgery and fixed edgewise orthodontic treatment.

Thus, the present study aimed to develop AI systems that predict the 3-D facial topography after orthognathic surgery and fixed edgewise orthodontic treatment based on the combination of GMM and deep learning. The usefulness of the combination of GMM and deep leaning was also discussed when predicting the 3-D shape changes in relation to treatment in the present study.

## Results

### Hard-tissue and soft-tissue changes after treatment

**Figure 1:**
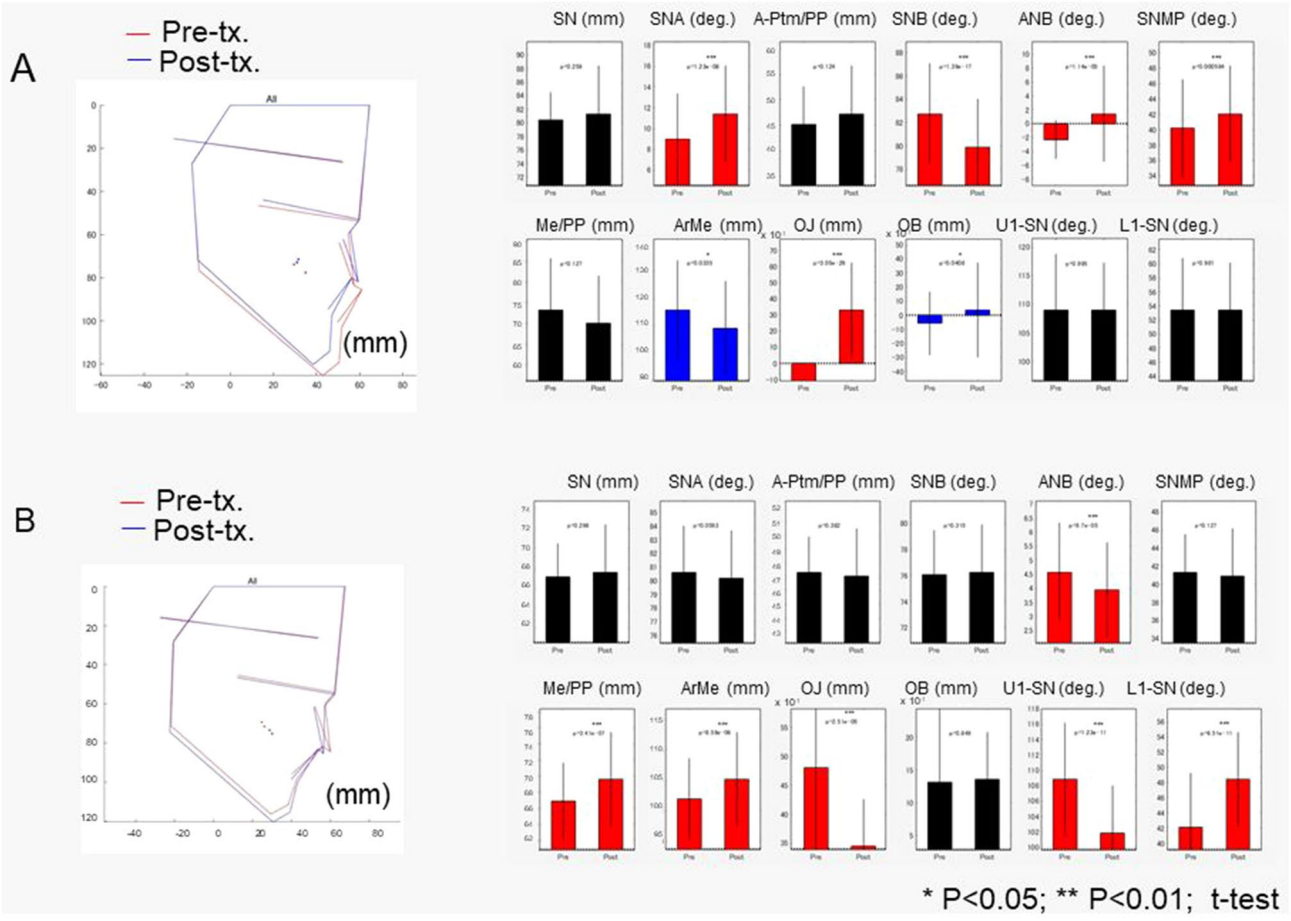
The average cephalometric changes in the Surgery (**A**) and Extraction (**B**) groups. The left column indicated the average cephalometric profilogram for pre-treatment (red) and post-treatment (blue). The right column shows the results of the comparison of cephalometric variables between pre-treatment and post-treatment. * Blue bars indicate significant differences (p < 0.05); *** red bars indicated significant differences (p < 0.001). SN indicates the distance between Sella and Nasion; SNA, Sella-Nasion-Point A angle; A-Ptm/PP, Point A to Ptm distance projected on the palatal plane; SNB, Sella-Nasion-Point B angle; ANB, Point A-Naion-Point B angle; SNMP, angle formed by SN and the mandibular plane; Me/PP, anterior lower face height; Ar-Me, distance between Articulare and Menton; OJ, overjet; OB, overbite; U1-SN, angle formed by upper incisors and the SN plane; L1-SN, angle formed by lower incisors and the SN plane.

**Figure 2:**
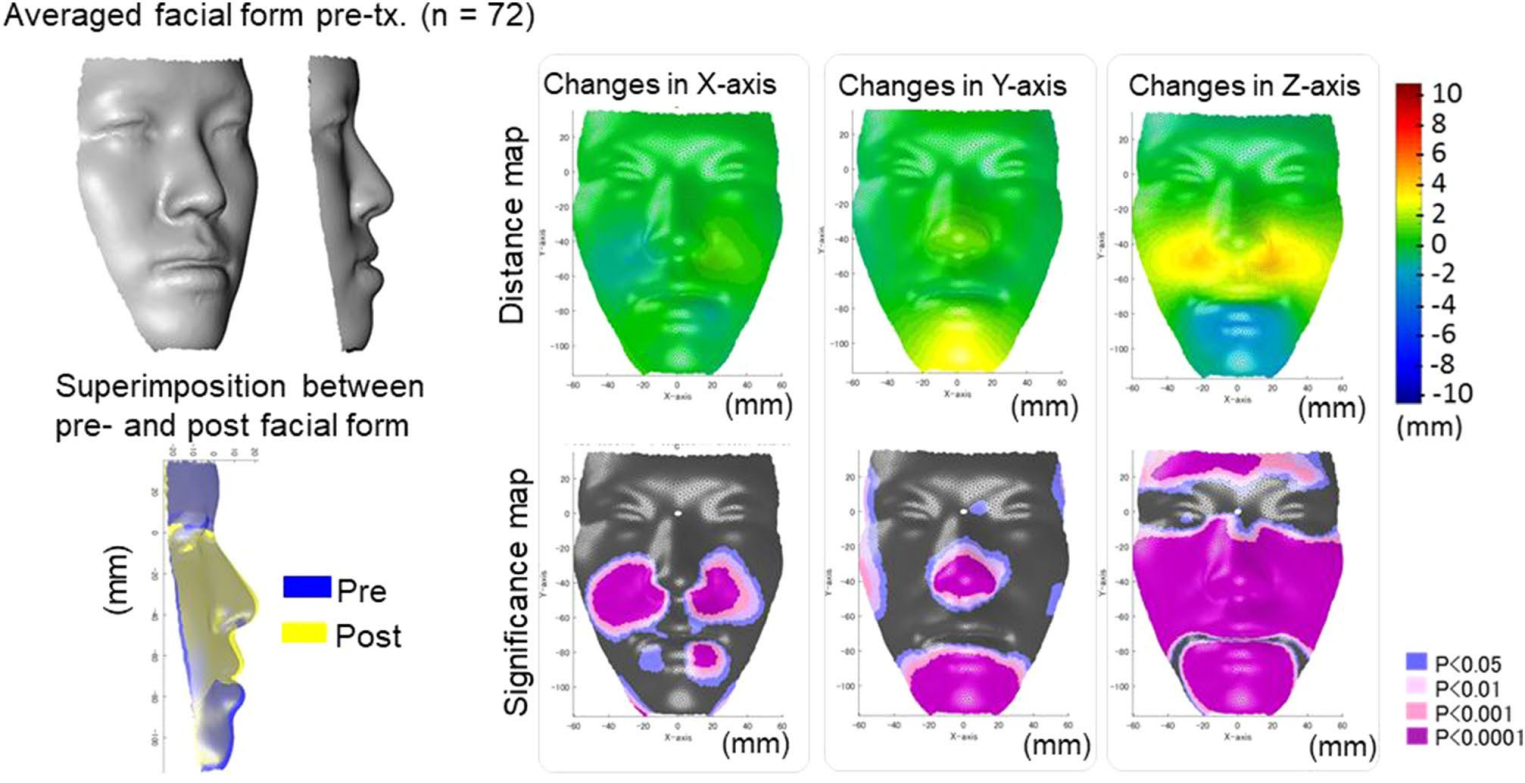
The average actual facial changes in the Surgery group for pre-treatment (top-left) and the superimposition of the actual facial changes between pre-treatment (blue) and post-treatment (yellow). For the significance probability maps, significant changes between pre-treatment and post-treatment are indicated as follows: blue, P < 0.05; pale pink, P < 0.01; dark pink, P < 0.001; purple, P < 0.000. For the distance maps, yellow indicates that the treatment changed from pre-treatment to post-treatment (post-treatment minus pre-treatment is a positive value, whereas blue indicates that the change had a negative value. Thus, in the horizontal direction in the Surgery group, yellow in the left-half face indicates lateral displacement and blue in the right-half face also indicates lateral displacement after treatment. In the vertical direction, blue indicates a downward displacement after treatment in the Surgery group and yellow indicates upward displacement after treatment. In the anteroposterior direction, yellow indicates a protrusive movement, whereas blue indicates a retrusive movement after treatment. The figures were created using a custom MATLAB software (MATLAB 2020a, The Math Works, Natick, MA, USA).

**Figure 3:**
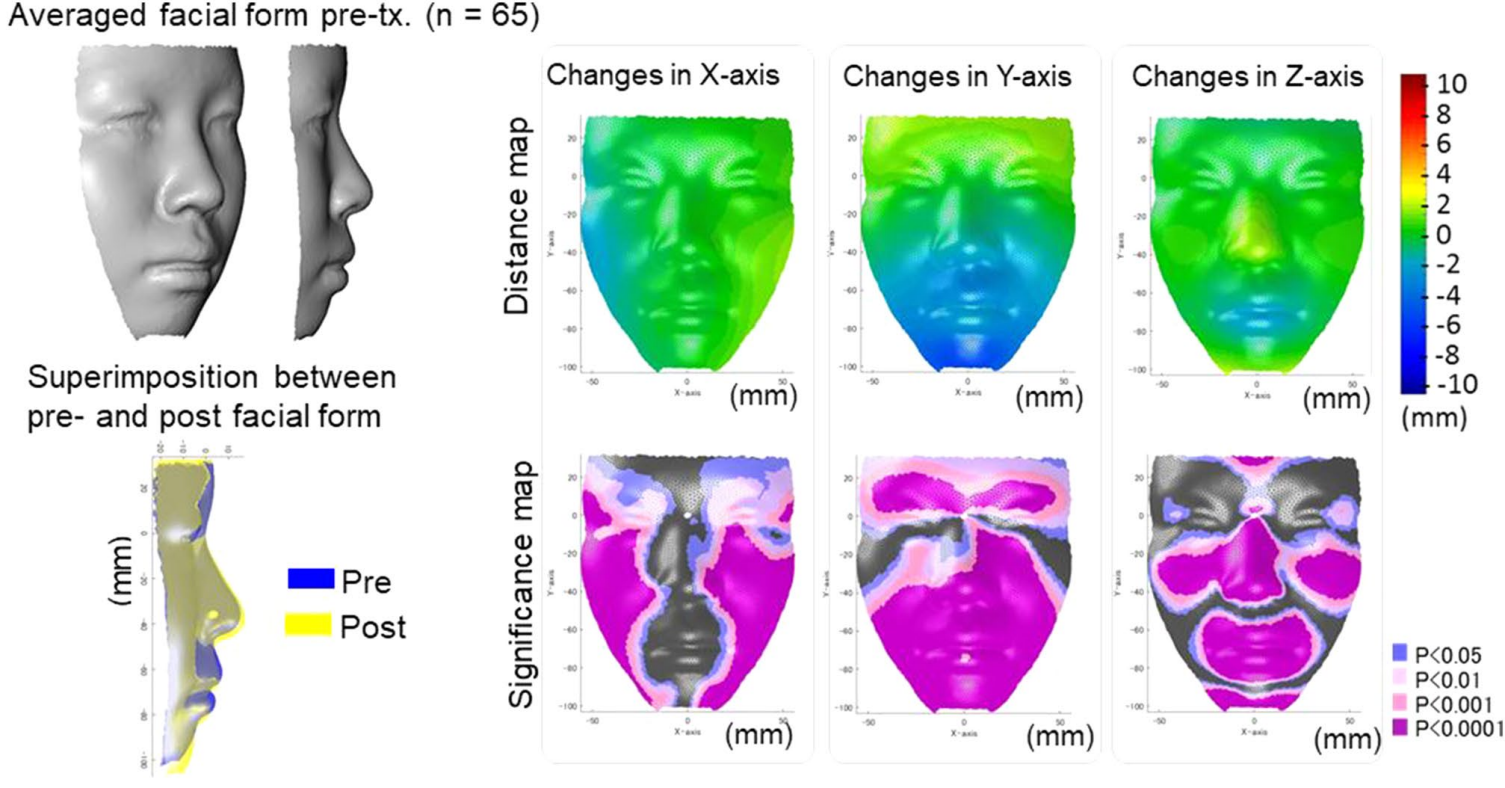
The average actual facial changes in the Extraction group for pre-treatment (top-left) and the superimposition of the actual facial changes between pre-treatment (blue) and post-treatment (yellow). For the significance probability maps, significant changes between pre-treatment and post-treatment are indicated as follows: blue, P < 0.05; pale pink, P < 0.01; dark pink, P < 0.001; purple, P < 0.0001. For the distance maps, yellow indicates that the treatment changes from pre-treatment to post- treatment (post-treatment minus pre-treatment is a positive value, whereas blue indicates that the change is a negative value. Thus, in the horizontal direction in the Extraction group, yellow in the left-half face indicates lateral displacement and blue in the right-half face also indicates lateral displacement after treatment. In the vertical direction, blue indicates downward displacement after treatment in the Extraction group and yellow indicates upward displacement after treatment. In the anteroposterior direction, yellow indicates a protrusive movement, whereas blue indicates a retrusive movement after treatment. The figures were created using a custom MATLAB software (MATLAB 2020a, The Math Works, Natick, MA, USA).

The changes of the hard and soft-tissue after treatment are shown in Figs. 1, 2 and 3. In the Surgery group, the maxillary positions were repositioned anteriorly by the average maxillary length of 2 mm, and the mandibular positions were repositioned posteriorly by two-jaw surgeries by the average mandibular effective length of 5 mm, leading to a significant increase in overjet with average changes of − 1 mm to + 3 mm. As the facial soft-tissue changes, the nasal alar and mid-facial cheek moved laterally by an average of 2 mm and the corner of the lip was moved mesially by an average of 1 mm (x-axis). As for the vertical changes (y-axis), the nose and chin were repositioned into superior positions by average distances of 2 mm and 3 mm, respectively, whereas the cheek was moved downward by the average distances of 1–2 mm. With regard to the antero-posterior changes (z-axis), the midface was moved forward by an average distance of 2 to 3 mm and the chin was moved backward by an average distance of 4 to 5 mm. Since the backward movement of the mandible was 5 mm, the soft-tissue movement corresponded well to the hard-tissue in the antero-posterior direction on the midline; however, the lateral and vertical changes seem to be difficult to predict from the assumption that the amount of movement of hard and soft-tissue has a proportional relationship.

In the Extraction group, the maxillary incisors were inclined lingually by an average of 6.5 degrees and the tip of the maxillary incisors was repositioned lingually by an average of 5 mm. The mandibular incisors were inclined lingually by an average of 6.2 degrees, and repositioned lingually by an average of 2.5 mm. As a result, the overjet was significantly decreased from + 4.8 mm to an average of + 3.3 mm. The patients showed significant growth in the mandibular effective length (5 mm) and lower facial height (3 mm), leading to a significant 0.5 degrees decrease in the A-N-B angle. As the facial soft-tissue changes, the facial width was increased laterally by an average of 2 mm (x-axis). As for the vertical changes (y-axis), the forehead was moved upward by 2 mm, the chin moved downward by 3 mm in average, which were corresponded to the facial growth. Regarding the antero-posterior changes (z-axis), the cheek, nose, and chin moved forward by 2 to 3 mm. The upper and lower lips were moved backward by average distances of 2 mm and 3 mm, respectively. As the upper and lower incisors positions were posteriorly moved by 5 mm and 2.5 mm, this upper and lower lip movement showed that the assumption that the amount of movement of hard-tissue and soft-tissue has a proportional relationship that is not applicable to post-treatment facial form.

### The development of the AI systems

**Figure 4:**
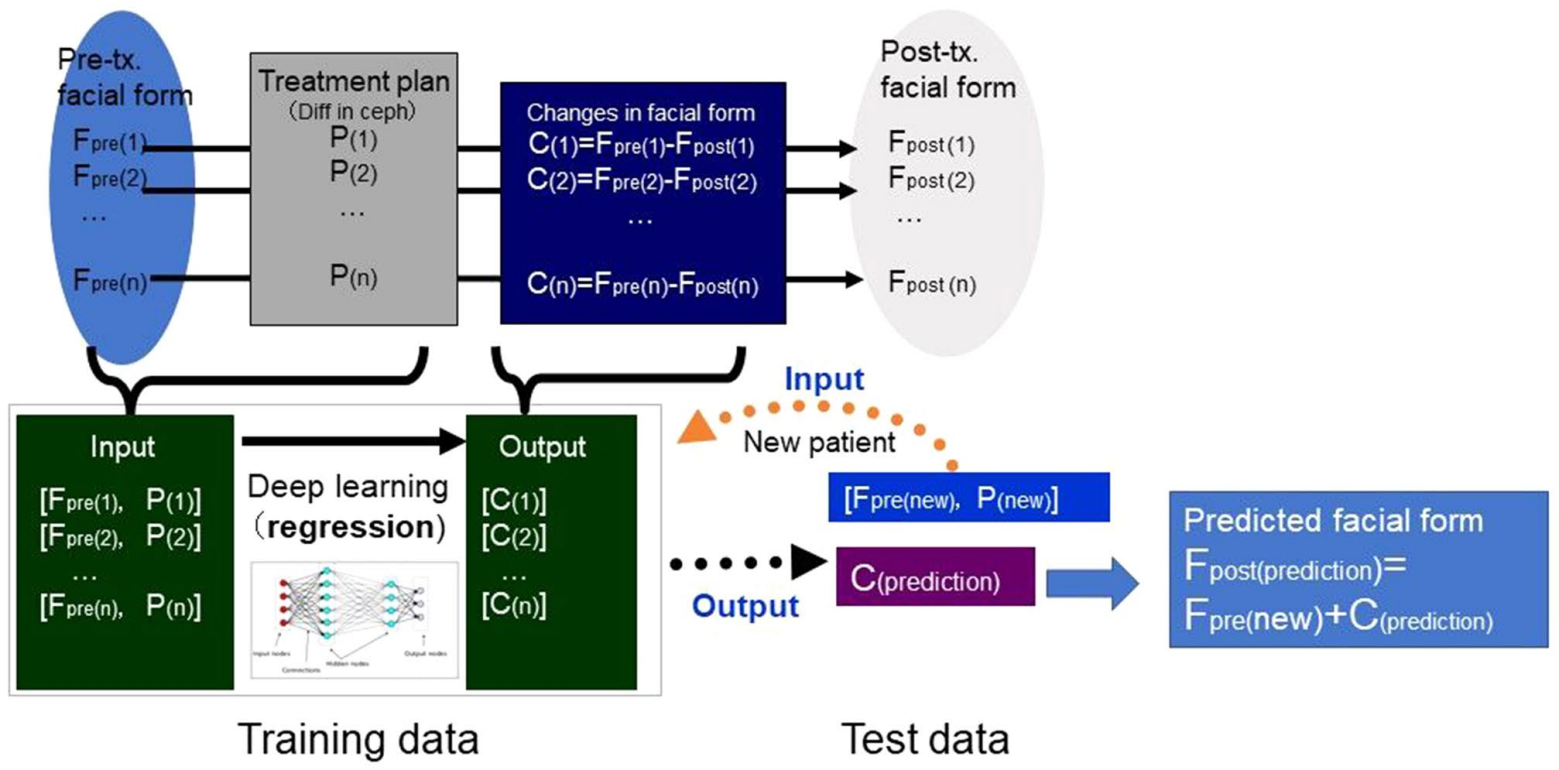
The mathematical model used to develop the artificial intelligence (AI) systems.

**Figure 5:**
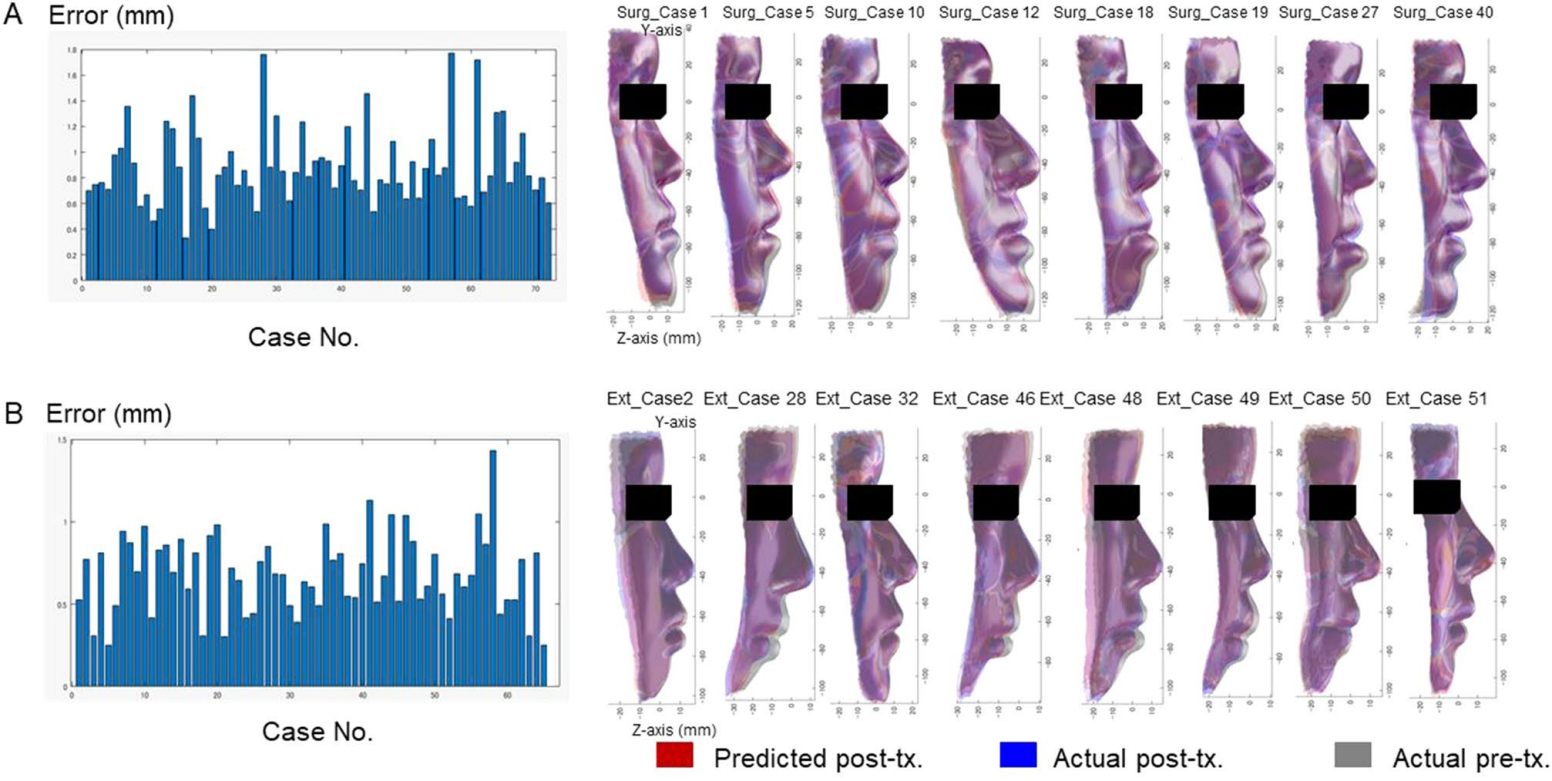
Average error for each patient with Systems S (**A**) and E (**B**). In the left column, the x-axis indicates the case number and the y-axis indicates the prediction error, which was defined as the difference between the predicted and actual post-treatment surface values. The right column shows the predicted face (red), actual post-treatment face (blue), and actual pre-treatment face (grey) for the eight representative cases in each group.

**Figure 6:**
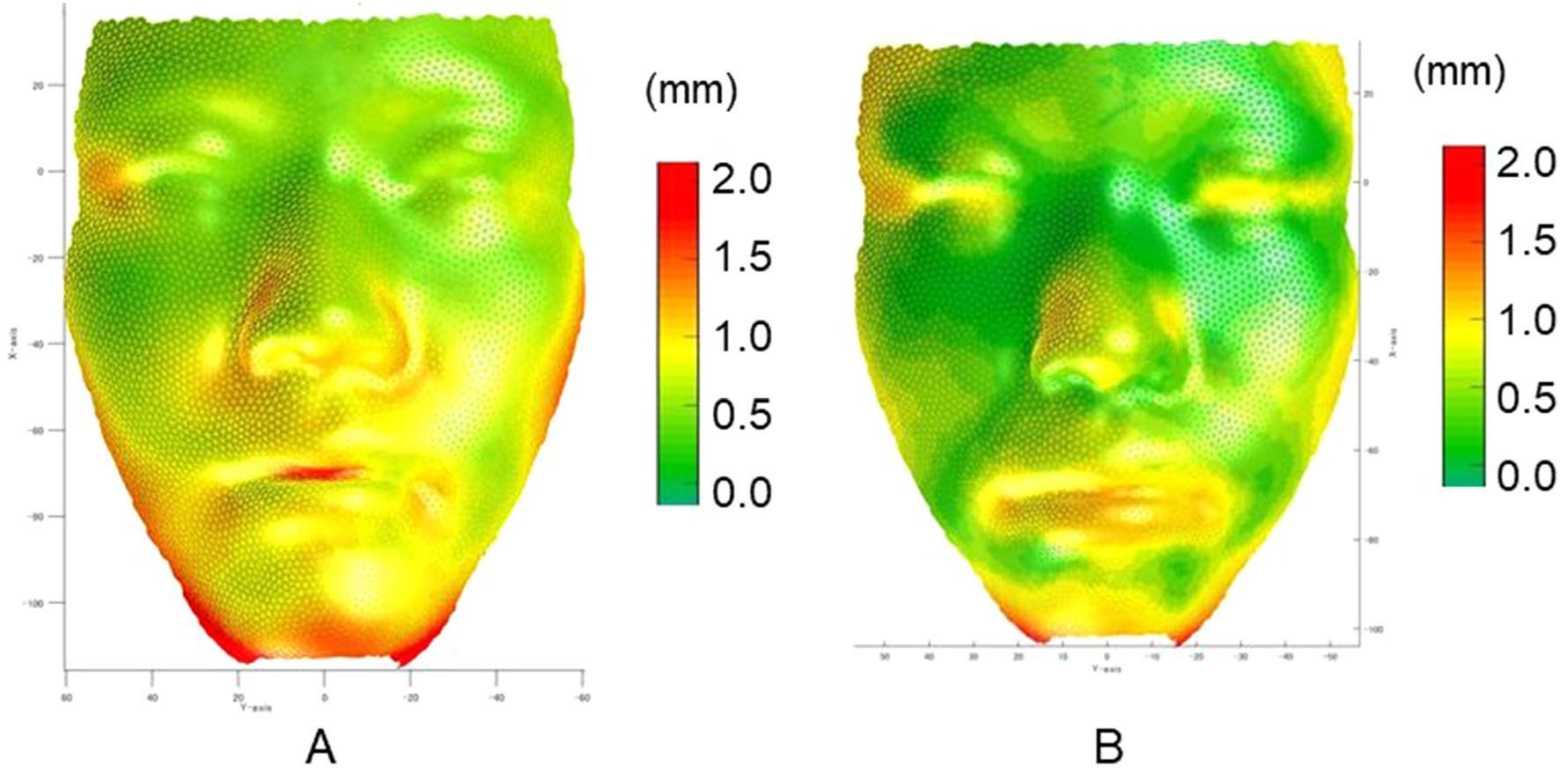
Average error (absolute differences in Z-axis between the predicted and actual facial form at post-treatment) with Systems S (**A**) and E (**B**).

**Figure 7:**
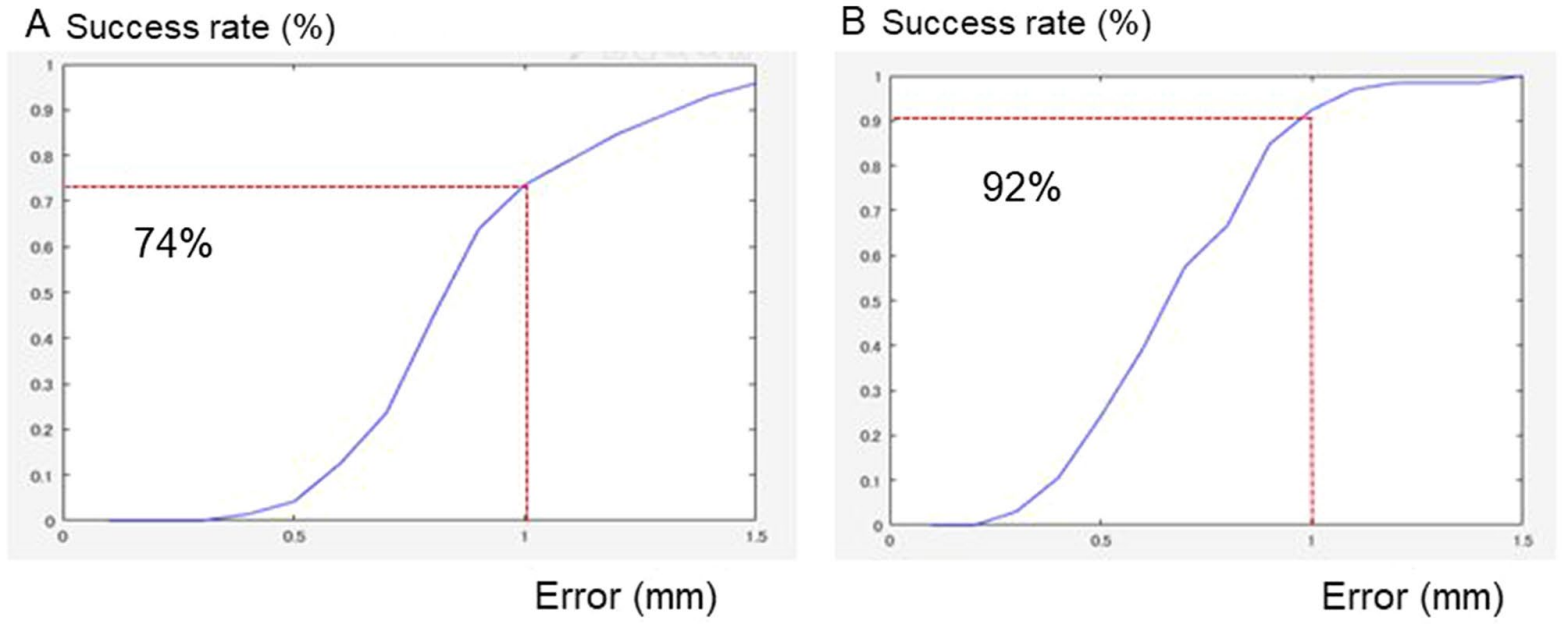
Success rate for each threshold between 0.1 mm and 1.5 mm for Systems S (**A**) and E (**B**).

Our system to predict facial morphology after treatment was successfully developed with a combination of GMM and deep leaning (Fig. 4). The prediction errors were demonstrated in Fig. 5. The system error was 0.89 ± 0.30 mm (S) and 0.69 ± 0.18 mm (E; Table 1). Maximum errors were observed in the nasal alar (S), the chin (S), the corner of the mouth (S), and the lower lip (S and E; Figs. 5 and 6). The total success rate of < 1 mm was 82% (74% [S] and 92% [E]), while the total success rate of < 2 mm was 100% (Fig. 7).

**Table 1:** Average error, standard deviation (S.D.), minimum and maximum values for Systems S and E.

(unit, mm)	Total error	Variation among the points (AveEachPc)			Variation among the patients (AveEachPt)		
		S.D	Min	Max	S.D	Min	Max
System S	0.89	0.36	0.42	4.02	0.30	0.33	1.77
System E	0.69	0.22	0.24	1.77	0.18	0.30	1.02

For the definition of the AveEachPc and AveEachPt, please see Supplementary Table S1.

## Discussion

In the present study, we successfully developed two AI systems that predict the 3-D facial shape after orthodontic treatment as well as orthognathic surgery by the combination of deep leaning and a GMM, which was a novel method for predicting 3-D shapes. Because this method is versatile, the present study suggests possibilities for developing systems that predicts any 3-D shape (e.g., breast shape) after any intervention or change (e.g., aging, cancer surgery, cosmetic surgery) once data are stored.

The proposed AI system to predict the facial form after orthognathic surgery (System S) showed an error of 0.89 ± 0.30 mm, which was small enough for clinical application. It is difficult to compare the present system directly with several commercial 3-D systems that are available for orthognathic surgery (e.g., the Dolphin system, Mimics, or other systems). However, studies^32,33^ demonstrated that the Dolphin 3-D software program had limited accuracy, especially in the upper lip and base of the nose. In a previous study^32^, the mean linear prediction error was 2.91 ± 2.16 mm and the mean error at the nasolabial angle was 8.1 ± 5.68 degrees. Notably, in most cases, the cheek around the nasal alar and the upper lip showed errors of > 5 mm. In contrast, a study using Dolphin 3-D software program showed errors that were, overall, clinically acceptable; however, the largest difference was also observed in the perioral area^35^. As for the facial changes after maxillary advancement osteotomy^36^, the differences in the mean absolute distance between the actual soft-tissue and the prediction were significantly smaller than 3 mm for all segmented anatomical areas, and ranged from 0.65 mm (chin) to 1.17 mm (upper lip). A study^37^ examining the changes of the malar–midfacial region after LeFort I osteotomy indicated that maxillary advancement leads to a more pronounced shifting of the soft-tissues in the malar-midfacial area in comparison to the upper lip.

There were also several attempts that studies that have aimed to predict 3-D soft-tissue outcomes based on the soft-tissue density on cone beam computed tomography^38–40^. Further, novel methods have been considered, such as the finite element model^40^ or anatomically driven soft-tissue simulation using a muscle template^41^. In an attempt to find a linear relationship between the soft-tissue and hard-tissue changes, it was impossible to find any specific proportional constant values in mandibular landmarks, and a wide range of relationships were observed between the soft-tissue and hard-tissue changes^38–40^. The nonlinear response of maxillary soft-tissues to underlying hard-tissue change in the nasal base and maxillary spline indicated the need for the use of nonlinear ratios for selected maxillary soft-tissue to hard-tissue movements in a simulation software program^39^. In the present study, we created an AI system that predicted the facial form after orthognathic surgery using nonlinear ratios and succeeded in predicting the post-treatment facial form, including malar-midfacial area.

The nasal alar, the chin, and the corner of the mouth showed errors of approximately 1.3 mm, whereas the lower lip showed an error of approximately 0.8 mm on average in system S. When compared to the commercial software program^32,37^ which showed errors of > 5 mm in the cheek around the nasal alar, the cheek was an area that commonly showed errors among the systems. When observing the soft-tissue changes in the present study, the nasal alar and mid-facial cheek were expanded laterally, moved downward, and moved forward, which was a new result that the previous studies did not find. These soft-tissue changes were considered to be one of the reasons why the cheek showed greater errors among the systems. However, our system well for this complicated movement and showed smaller errors (1.3 mm) in comparison to the commercial software program (> 5 mm). Since the mid-facial deficiency or cheek concavity is an important factor when considering the advancement of the maxilla by LeFort I, the present study is useful when deciding the amount of advancement.

The corner of the mouth is another area that has not previously been confirmed. In the present study the corner of the mouth showed significant mesial movement after orthognathic surgery, which means that the mouth width became narrower. This may be related to the assumption that backward movement of the lower lip may reduce the lip prominence from the axial view, which may reduce the width of the mouth. The prediction for the corner of the mouth also showed a greater error, which means that the hard-tissue and soft-tissue movement in the corner of the mouth is complex.

Because there were no 3-D systems that could predict the 3-D facial form after orthodontic treatment with four premolar extraction, it was difficult to compare the present AI system (System E) to previous studies. However, the 2-D Dolphin system^26^ showed a mean error of 2.02 mm (tip of the nose) to 4.13 mm (soft-tissue gnathion) in the 2-D system; thus, the error of the present system, which was 0.69 mm, was considered to be small enough for clinical application. When the clinically acceptable prediction error was set as 2 mm, a previous study using 2-D systems showed success rates of 47.71% and 54.90%^23^; in contrast, the present system showed a success rate of 100%.

Regarding the facial portions showing prediction errors in system E, the lower lip showed a greater error (approximately 1.1 mm) in comparison to the upper lip (0.8 mm) in the present study. This result partially corresponded to the prediction error observed in a 2-D system^26^. The 2-D system^26^ showed an error of 1.59 mm for the upper lip, while the error of the lower lip was 2.77 mm. In contrast, another study^19^ showed that changes in the lower lip in response to orthodontic tooth movement were more predictable than those of the upper lip. Another study examining the lip changes in relation to the lip strain, which were expressed as lip thickness^42^, showed a strong correlation found between osseous changes and soft-tissue changes in subjects with thin lips (r = 0.9), whereas no significant correlation was found in subjects with thick lips (r = 0.48–0.67). This result indicated that pre-treatment functional morphological structures influence lip changes. It has been shown that the value of the proportionality constant in the lip region varies greatly depending on the case. A study examining two different types of malocclusion (i.e., maxillary protrusion and bimaxillary protrusion cases) showed that the cases with maxillary protrusion showed slightly less soft-tissue change in comparison to cases with bimaxillary protrusion^5^. The relationship between hard-tissue and soft-tissue movement is too complex to examine in a relatively simplistic way^20^. Because the method that the present system employed involved prediction based on past cases in patients with a similar facial form, we considered that the present system could predict more complicated movement in comparison to the conventional assumption that the proportional relationship between hard-tissue and soft-tissues can be applied to all patients.

The treatment results that we employed to develop the AI system were obtained from the records of two hospitals that had similar treatment procedures; thus, the system can mimic treatment results from these hospitals. However, it is unknown whether these systems can be applied in other hospitals or clinics. Further studies are needed to validate these results.

## Conclusion

The combination of GMM and deep learning were confirmed to useful for the development of AI systems that predict the 3-D facial topography after orthognathic surgery (S) and fixed edgewise orthodontic treatment with four premolar extraction (E). The system error for two systems was 0.94 ± 0.43 mm (S) and 0.69 ± 0.28 mm (E), respectively. Maximum errors of approximately 0.8–1.2 mm were observed in the nasal ala, chin, corner of the mouth (S) and the lower lip (E). Although these difficult areas were clinically important regions, the total success rate at < 1 mm was 81% (54% [S] and 98% [E]); and that at < 2 mm was 100%, which indicates the possibility of applying the system in the clinical setting.

## Material and methods

### Subjects

This was a retrospective study in a secondary adult care setting. A total of 137 pre-treatment and post-treatment records from patients who underwent orthognathic surgery (Surgery group; n = 72; mean age = 23.5 years) and those who underwent orthodontic treatment with premolar extraction (Extraction group; n = 65; mean age = 15.6 years) were enrolled. The inclusion criteria for the Surgery group were as follows: pre-treatment patients who visited the orthodontic department of Osaka University Dental Hospital, during 2011–2015; age 15–37 years; negative overjet; no severe mandibular deviation of > 5 mm; planned orthognathic surgery with LeFort I osteotomy and bilateral sagittal split osteotomy; no facial paralysis; body mass index, 18.50–24.99; and no maxillofacial plastic surgery in the previous six months. The inclusion criteria for the Extraction group were as follows: pre-treatment patients who visited the orthodontic department of Osaka University Dental Hospital and Kanomi Dental Hospital, during 2011–2015; age 12–37 years; positive overjet; no severe mandibular deviation of > 5 mm; planned edgewise orthodontic treatment with four premolar extraction; no facial paralysis; body mass index, 18.50–24.99; and no maxillofacial plastic surgery in the previous six months. A written informed consent form was distributed to and signed by all participants. Informed consent was approved by the Research Ethics Committee, Osaka University Dental Hospital (project ID: H25-E37-1). The treatment outcome was assessed in 2014–2020, after treatment was completed. The sample size was decided based on practical grounds (existing study cohort). There were no missing data on the predictors or outcomes. All experiments were performed in accordance with the relevant guidelines and regulations, and all experimental protocols were approved by the Research Ethics Committee, Osaka University Dental Hospital. Further, informed consent was obtained to publish the images in an online open access publication.

### Data acquisition

Facial images of the subjects at rest were recorded once with a 3-D image capturing device (3-DMDcranial System, 3-DMD, Atlanta, GA, USA) with a natural head position without head support. 3-D images were acquired after treatment during the retention period.

### Data processing

#### Landmark identification

For each 3-D facial image, the positions of 18 landmarks (nasion, pronasale, subnasale, labiale superious, stomion, labiale inferious, submentale, pogonion, porion [right and left], exocanthion [right and left], endocanthion [right and left], alar curvature [right and left], and cheilion [right and left]^43^) were identified and digitized using a commercial software program (Face Rugle, Medic Engineering Co., Kyoto, Japan). Facial images were standardized with a common coordinate system [Supplementary Fig S1]^44^.

### Wire mesh fitting

For each participant at each time point, a landmark-based GMM analysis^45^ was performed using the HBM software program (National Institute of Advanced Industrial Science and Technology, Japan). This analysis generated a fitted mesh consisting of a set of 6017 points as nodes (Supplementary Fig. S2).

### Morphological differences between pre-treatment and post-treatment

To determine morphological characteristics of the facial soft-tissue surface changes during treatment in the Surgery and Extraction groups, the coordinate values of each node of the wire mesh on the facial surface on the x, y, and z axes were statistically analyzed. A one-sample *t*-test was performed to compare each axis in the two subject groups. To visualize the differences between pre-treatment and post-treatment in the two subject groups, the results were represented as a color map showing the P values for the comparison between the two subject groups (the significance probability map) and a color map representing the differences between two subject groups (hereafter referred to as the distance map)^45^. P values of < 0.05 were considered to indicate statistical significance.

### AI systems

Based on the deep learning method, we developed two AI systems (S and E) to predict changes in the coordinate values of the semi-landmarks due to orthognathic surgery (Surgery group) and orthodontic treatment with four premolar extraction (Extraction Group), respectively. As predictive variables for the AI system, the x-, y- and z-coordinate values of the 6017 3-D facial semi-landmarks for the pre-treatment, x- and y-coordinate values of 27 cephalometric landmarks (Table 1), and x- and y-coordinate changes during the treatment of 16 cephalometric landmarks (Table 1) were employed (i.e., 6,017 × 3 + 27 × 2 + 16 × 2 = 18,137 dimensions). The AI system’s predicted outcome was set as the predicted change for each semi-landmark on the 3-D face (6,017 × 3 = 18,051 dimensions). The predicted post-treatment facial morphology was calculated as the sum of the coordinate value on the face before treatment and the predicted change for each semi-landmark (Fig. 4).

### Structure of deep neural networks

The number of neurons in the input layer is the same as the number of features in our data. In the present study, 18,137 neurons were set as input layers. The 18,051 neurons were set for the regression output layer, since the expected output was the predicted change for each semi-landmark on the 3-D face. The system used two dense layers and one dropout layer, in which dense layers were the most common type of layer, where each of its neurons was connected to the neurons of the previous and next layer. Each dense layer has an activation function that determines the output of its neurons based on the inputs and weights of the synapses. In the present study, Rectified Linear Unit (ReLU) activation was performed for each dense layer. Dropout layers were also utilized for regularization, which randomly dropped some of the input units to 0, which helps to reduce the chance of overfitting the neural network. In our system, the dropout layer was set as 30%. As the optimized layer, which is the optimizing algorithm that helps achieve better results for the loss function, the Adam optimizer was used for learning. The mean squared error was set as the loss function when compiling. The system was trained with 30 epochs using keras and TensorFlow libraries in Python, where we intentionally stopped the training process when the validation loss did not improve for certain epochs. Here, to tune the model's hyperparameters, we used records from five patients who were excluded from our samples (two upper premolar extraction and one jaw surgery). As a result, the same hyperparameters were used for both systems. Calculation was conducted using an Octopus supercomputer at Osaka University. Once the system was trained, the predicted change for each semi-landmark on the 3-D face for the new patients was estimated using the trained neural networks.

### Evaluation

The performance of each AI system was evaluated using 11-fold cross-validation. Namely, the whole data were randomly split into eleven sets (or folds). The following procedure was conducted for each of the eleven folds: a model was trained using the 10 folds as training data; the resulting model was validated with the remaining data; and the system error for all patients was computed in the eleven folds. Here, the system error was determined as the difference between actual post- and predicted post-treatment coordinates of the semi-landmarks in the Z-axis (Table 1). Furthermore, the total success rate was examined, wherein successful cases were defined as those with an average error of < 1 mm or < 2 mm.

## Supplementary Information

Supplementary Information.
