# Supplementary material for: Development of novel artificial intelligence systems to predict facial morphology after orthognathic surgery and orthodontic treatment in Japanese patients
Source: Sci Rep. 2021 Aug 4;11:15853. doi: 10.1038/s41598-021-95002-w (PMC8339122; doi:10.1038/s41598-021-95002-w)
Supplement: Supplementary file 1 — Supplementary Information. [file 41598_2021_95002_MOESM1_ESM.docx]

**Supporting Files**

**Development of novel artificial intelligence systems to predict facial morphology after orthognathic surgery and orthodontic treatment in Japanese patients**

**Supplementary Table S1.** Calculation of the average error (AveEachPt, AveEachPc). *d(i,j)* indicates the error of facial point j in patient i.

|  | **No. of semi-landmarks (nodes of the wire mesh)** | | | | | | **Average** |
| --- | --- | --- | --- | --- | --- | --- | --- |
| **Patient** | *d(1,1)* | *d(1,2)* | *…* | *d(1,j)* | *…* | *d(1,6017)* | **AveEachPc(*i*=1)** |
|  | *d(2,1)* |  |  | *d(2,ｊ)* |  | *d(2,6017)* | **AveEachPc(*i*=2)** |
|  | *…* |  |  | *…* |  | *…* | **…** |
|  |  |  |  |  |  |  |  |
|  | *d(i,1)* | *d(i,2)* | *…* | *d(i,j)* | *…* | *d(i,6017)* | **AveEachPc(*i*)** |
|  |  |  |  | *…* |  |  |  |
|  | *…* |  |  |  |  | *…* | **…** |
|  | *d(n,1)* | *d(n,2)* | *…* | *d(n,j)* | *…* | *d(n,6017)* | **AveEachPc(*i*=6017)** |
| **Average** | **AveEachPt**  **(*j*=1)** | **AvePtEach**  **(*j*=2)** | **…** | **AveEachPt**  **(*j*)** | **…** | **AveEachPt**  **(*j*=n)** | **Total error** |

**
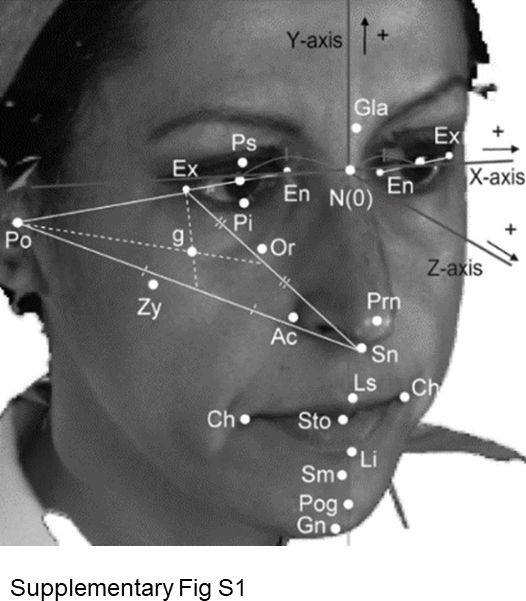
**

**Supplementary Fig S1.** The coordinate system ^1^. The 3D coordinate system. The nasion (N) was defined as the origin (O). The sagittal plane was defined as the plane passing through the origin and perpendicular to the line through the midpoint of the right exocanthion (Ex) and endocanthion (En), and the midpoint of the left Ex and En. The axial plane was defined as the plane passing through the origin and parallel to the line connecting the porion and the geometric center (g) of the porion (Po), subnasale (Sn), and Ex on the image projected onto the sagittal reference plane. The coronal plane was defined as the plane passing through the origin and perpendicular to both the axial and sagittal planes. Ac, alar curvature point; Prn, pronasale; Ls, labiale superious; Sto, stomion; Ch, cheilion; Li, labiale inferious; Sm, submentale; Pog, pogonion (cited from a reference ^1^ with permission.). Publication of identifying images in an online open-access publication was obtained.


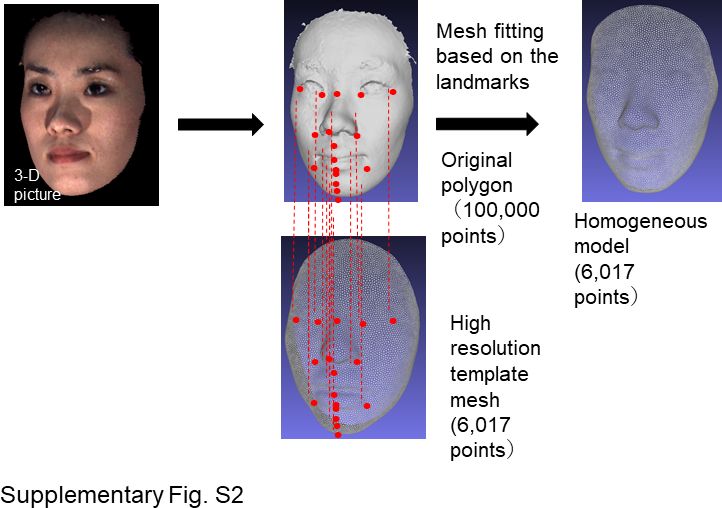


**Supplementary Fig. S2.** A schematic illustration of the wire mesh fitting (Homogeneous model cited from a reference ^2^ with permission). Publication of identifying images in an online open-access publication was obtained.

**References**

1 Tanikawa, C., Zere, E. & Takada, K. Sexual dimorphism in the facial morphology of adult humans: A three-dimensional analysis. *Homo* **67**, 23-49, doi:10.1016/j.jchb.2015.10.001 (2016).

2 Tanikawa, C., Akcam, M. O. & Takada, K. Quantifying faces three-dimensionally in orthodontic practice. *J Cranio Maxill Surg* **47**, 867-875, doi:10.1016/j.jcms.2019.02.012 (2019).
